# Commentary: Not too long, but long enough?

**DOI:** 10.1016/j.xjtc.2021.04.020

**Published:** 2021-04-27

**Authors:** Kenji Minakata

**Affiliations:** Division of Cardiovascular Surgery, Lewis Katz School of Medicine at Temple University, Philadelphia, Pa

**Figure:**
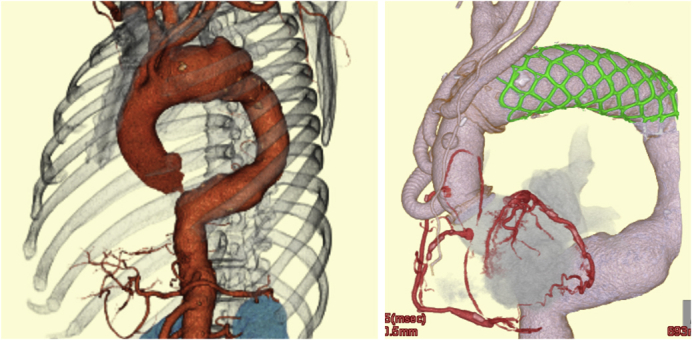
A 3D-CTA with preoperative and postoperative images of the aortic arch aneurysm treated with FET.

**Central Message**

The key to success with the FET technique is to use a graft of the appropriate size and adequate length to cover both the proximal and distal ends of the distal arch/proximal descending aneurysm.

See Article page 33.

The frozen elephant trunk (FET) technique has been widely accepted as an attractive adjunct to treat extended distal aortic arch/proximal descending thoracic aortic aneurysms. In this issue of the *Journal*, Wakabayashi and colleagues^1^ report a very interesting case focusing on a technical aspect of transapical thoracic endovascular aortic repair (TEVAR) in a patient with a residual nondissecting distal aortic arch aneurysm along with extensive atherosclerosis. The initial procedure, which was performed before the current procedure, was open total aortic arch replacement using the FET technique, including debranching of all 3 arch vessels. According to the initial postoperative course and the computed tomography (CT) findings, the stent graft used for FET was not long enough to cover the distal end of the aneurysm, causing a lack of distal sealing.

Given the extensive atherosclerotic changes in the distal descending thoracic aorta with sigmoid configuration, a retrograde approach with an endovascular technique to address this aneurysm appeared to be prohibitive. Therefore, a decision was made to proceed with an antegrade transapical TEVAR to cover the residual distal arch/proximal descending thoracic aortic aneurysm under extracorporeal membrane oxygenator (ECMO) support.

Despite the successful results, this case raises some questions. First, at the initial operation, more precise measurement of the aneurysm and adequate choice of the length of the FET graft could have made the additional procedure (the present procedure) unnecessary. Obviously, care must be taken not to insert the FET graft too distally, which can cause spinal cord ischemia and/or increase the risk of thromboembolism. This should never be intended to be 2-stage operation unless it is unavoidable. In addition, at the initial operation, the proximal anastomosis was made at the level of the proximal aortic arch (zone 0). This might have made the FET graft too short and might have made it more difficult to insert the FET graft sufficiently distal to cover the distal end of the aneurysm. Therefore, the distal anastomosis should have been made at least on zone 1, preferably on zone 2.

In this very difficult situation, an antegrade transapical approach was perhaps the only safe bailout solution. We are now more familiar with this approach from the recent experience with transapical transcatheter aortic valve replacement. The authors used ECMO support during the TEVAR for hemodynamic stability and prevention of cerebral malperfusion, which definitely led to their excellent results. Second, according to the presented 3-dimensional CT angiogram, the patient still appeared to have a distal descending thoracic aortic aneurysm at the level near the diaphragm, which could become problematic in the near future. Thus, devising a total management strategy for the whole diseased aorta at the very beginning of the presentation is important to minimize the invasiveness and number of procedures.

## References

[1] Wakabayashi N., Kikuchi Y., Shibagaki K., Kamiya H. (2021). Transapical thoracic endovascular aortic repair with a frozen elephant trunk for thoracic aortic aneurysm with shaggy aorta. J Thorac Cardiovasc Surg Tech.

